# Hydrothermal Growth of an Al-Doped α-Ga_2_O_3_ Nanorod Array and Its Application in Self-Powered Solar-Blind UV Photodetection Based on a Photoelectrochemical Cell

**DOI:** 10.3390/mi14071336

**Published:** 2023-06-29

**Authors:** Jing-Chun Guo, Guang-Wu Sun, Ming-Ming Fan, Xu-Cheng Fu, Jia-Jia Yao, Yu-Dong Wang

**Affiliations:** 1 Department of Experimental and Practical Teaching Management, West Anhui University, Lu’an 237012, China; 2 Mechanical and Electrical Engineering College, Hainan Vocational University of Science and Technology, Haikou 571126, China; 3 College of Physics, Taiyuan University of Technology, Taiyuan 030024, China; 4 College of Biological and Chemical Engineering, Guangxi University of Science and Technology, Liuzhou 545006, China

**Keywords:** hydrothermal growth, Al-doped α-Ga_2_O_3_ nanorod array, self-powered, solar-blind UV photodetection, photoelectrochemical cell

## Abstract

Herein, we successfully fabricated an Al-doped α-Ga_2_O_3_ nanorod array on FTO using the hydrothermal and post-annealing processes. To the best of our knowledge, it is the first time that an Al-doped α-Ga_2_O_3_ nanorod array on FTO has been realized via a much simpler and cheaper way than that based on metal–organic chemical vapor deposition, magnetron sputtering, molecular beam epitaxy, and pulsed laser deposition. And, a self-powered Al-doped α-Ga_2_O_3_ nanorod array/FTO photodetector was also realized as a photoanode at 0 V (vs. Ag/AgCl) in a photoelectrochemical (PEC) cell, showing a peak responsivity of 1.46 mA/W at 260 nm. The response speed of the Al-doped device was 0.421 s for rise time, and 0.139 s for decay time under solar-blind UV (260 nm) illumination. Compared with the undoped device, the responsivity of the Al-doped device was ~5.84 times larger, and the response speed was relatively faster. When increasing the biases from 0 V to 1 V, the responsivity, quantum efficiency, and detectivity of the Al-doped device were enhanced from 1.46 mA/W to 2.02 mA/W, from ~0.7% to ~0.96%, and from ~6 × 10^9^ Jones to ~1 × 10^10^ Jones, respectively, due to the enlarged depletion region. Therefore, Al doping may provide a route to enhance the self-powered photodetection performance of α-Ga_2_O_3_ nanorod arrays.

## 1. Introduction

Ga_2_O_3_ is a promising wide-bandgap oxide semiconductor for potential applications in solar-bind UV photodetection, high-power devices, gas sensors, and transparent conductive oxides [1,2,3,4,5]. Among these applications, solar-bind UV photodetection can be more simply and easily investigated, owing to its inherent solar-bind UV absorption properties, which can be applied in flame detection, UV astronomy and dosimetry, water purification, space-to-space communication, and missile warnings [1,2,3,4,5]. Therefore, large amounts of research have been performed on the basis of the MSM (metal–semiconductor–metal) structure [3,4,5]. However, the MSM structure always needs external biases to separate the photogenerated carriers. Schottky junctions, p–n junctions, or n–n junctions can realize self-powered photodetection, owing to their built-in electric field [6]. And, such a mode has caught much attention, owing to it working at 0 V without external biases, which possesses the advanced properties of energy conservation and environmental protection [6]. In fact, self-powered photodetectors have been realized among other metal oxide semiconductors such as TiO_2_, ZnO, CuO, and NiO [6,7]. As a reference for Ga_2_O_3_-based photodetectors, constructing Schottky junctions, p–n junctions, or n–n junctions are still the most feasible ways to realize self-powered photodetection.

Currently, element doping, such as Si, Ge, Sn, In, or Al doping, is an important technique to realize the more multifunctional applications mentioned above, due to its controllable technique of modulating the electrical and optical properties [8,9,10,11,12,13,14,15,16,17]. Unlike other elements, In or Al doping can more effectively realize an extension of the bandgap [8,9,10,11,12,13,14,15,16]. For example, the bandgap of inherent Ga_2_O_3_ can be extended into smaller values by alloying In with it [8,9,10,11], and can also be expanded into larger values by alloying Al with it [12,13,14,15,16]. Therefore, Al doping is more important for Ga_2_O_3_ to be applied in solar-bind UV photodetection, due to the fact that its solar-blind UV absorption characterization can be maintained. S.-H. Yuan et al. reported that the peak responsivity of an MSM photodetector at 230 nm, under a bias voltage of 5 V, can reach 53.61 times greater than that of a photodetector without doping any Al content [13]. And Q. Feng et al. obtained a 10-times-higher photocurrent for an (Al_0.12_Ga_0.88_)_2_O_3_ MSM device, compared to an undoped photodetector [12]. Moreover, a graphene/(AlGa)_2_O_3_/GaN self-powered device also showed excellent photodetection properties, with a peak responsivity of ∼20 mA/W, a rise time of ∼2 μs, and a decay time of ∼10 ms [14]. Therefore, Al-doped Ga_2_O_3_ can bring an improvement in photoresponsivity, both in a non-self-powered photodetection mode and in a self-powered photodetection mode [12,13,14,15,16]. According to the literature, many of the recent efforts to dope Al into Ga_2_O_3_ described above were performed using metal–organic chemical vapor deposition (MOCVD), magnetron sputtering, molecular beam epitaxy (MBE), and pulsed laser deposition (PLD) [12,13,14,15,16]. Obviously, these methods are expensive and complicated. However, an α-Ga_2_O_3_ nanorod array can be easily grown on FTO using the hydrothermal and post-annealing processes, which have the advantages of being more simple and much cheaper procedures [18,19,20]. To the best of our knowledge, such processes have not been applied for Al-doped α-Ga_2_O_3_ nanorod arrays. More importantly, α-Ga_2_O_3_ nanorod array/FTO can be employed as the basic structure for realizing self-powered photodetection, due to the fact that a Schottky junction can be formed at the surface between the α-Ga_2_O_3_ nanorod array and the electrolyte. This is a famous photodetection mode, when the α-Ga_2_O_3_ nanorod array/FTO structure is immersed into the electrolyte in a photoelectrochemical (PEC) cell [18,19,20,21,22,23]. Therefore, we used an Al-doped α-Ga_2_O_3_ nanorod array/FTO as the photoanode in a PEC cell to demonstrate the self-powered photodetection properties.

Herein, an Al-doped α-Ga_2_O_3_ nanorod array on FTO was achieved using the hydrothermal and post-annealing processes. It is the first time that an Al-doped α-Ga_2_O_3_ nanorod array on FTO has been realized using a much simpler and cheaper way. Based on a PEC cell, a self-powered Al-doped α-Ga_2_O_3_ nanorod array/FTO photodetector was demonstrated as the photoanode, with a peak responsivity of 1.46 mA/W at 260 nm at 0 V (vs. Ag/AgCl). Moreover, the device had a response speed of 0.421 s for rise time and 0.139 s for decay time under solar-blind UV (260 nm) illumination. The responsivity of the Al-doped device was ~5.84 times larger, and the response speed was relatively faster than the performance of an undoped device, which is due to the fact that a larger Schottky barrier and wider depletion region may exist in the Al-doped α-Ga_2_O_3_ nanorod array/FTO device than that in the undoped α-Ga_2_O_3_ nanorod array/FTO PEC device. Owing to the enlarged depletion region resulting from increasing the positive biases from 0 V to 1 V, the responsivity, quantum efficiency, and detectivity of the Al-doped device were respectively enhanced from 1.46 mA/W to 2.02 mA/W, from ~0.7% to ~0.96%, and from ~6 × 10^9^ Jones to ~1 × 10^10^ Jones.

## 2. Materials and Methods

During hydrothermal fabrication processes, gallium nitrate hydrate (Ga(NO_3_)_3_·xH_2_O) and aluminum nitrate hydrate (Al(NO_3_)_3_·9H_2_O) were selected as the Ga source and Al source, respectively. Washed FTO substrates were put into a hydrothermal reactor with a PTFE lining, half of which was immersed into the precursor solution. Before the undoped α-Ga_2_O_3_nanorod array and Al-doped α-Ga_2_O_3_ nanorod array were realized, an undoped GaOOH nanorod array and Al-doped GaOOH nanorod array were grown on FTO at 200 ℃ for 10 h through hydrothermal processes. During the hydrothermal growth of the Al-doped samples, gallium nitrate and aluminum nitrate aqueous solution mixed with a mole ratio of 1:0.1 for Ga and Al, and the solution was poured into the reactor as the precursor solution. For the undoped samples, the precursor solution only contained gallium nitrate aqueous solution. After the as-grown undoped GaOOH nanorod array/FTO and Al-doped GaOOH nanorod array/FTO samples were washed by ultrapure water and blown by dry N_2_ gas, they were post-annealed immediately at 550 °C in O_2_ (10 sccm) atmosphere for 2 h. Thus, the undoped α-Ga_2_O_3_ nanorod array and Al-doped α-Ga_2_O_3_ nanorod array were fabricated on FTO substrates. The material properties were investigated by scan electron microscopy (SEM), energy dispersive spectroscopy (EDS), and X-ray diffraction (XRD) with Cu K*α* as a radiation source and UV-vis diffuse reflectance absorption spectra.

An undoped α-Ga_2_O_3_ nanorod array/FTO and Al-doped α-Ga_2_O_3_ nanorod array/FTO structure were immersed into a 0.5 M Na_2_SO_4_ aqueous solution, and measured as photoanodes in a PEC cell. The undoped α-Ga_2_O_3_ nanorod array/FTO or Al-doped α-Ga_2_O_3_ nanorod array/FTO, Pt, Ag/AgCl were, respectively, used as the working electrode, the counter electrode and the reference electrode in a three-electrode system. The self-powered properties, including the dark current, light current and transient photoresponse, were investigated at 0 V (vs. Ag/AgCl) based on an electrochemical workstation. When the undoped α-Ga_2_O_3_ nanorod array/FTO or Al-doped α-Ga_2_O_3_ nanorod array/FTO, and Pt electrodes were connected with a lock-in amplifier, the spectral responsivity was also collected at 0 V (vs. Ag/AgCl) under illumination of the monochromatic light generated by a 300 W UV-enhanced Xe lamp and a monochromator.

## 3. Results and Discussion

**Figure 1:**
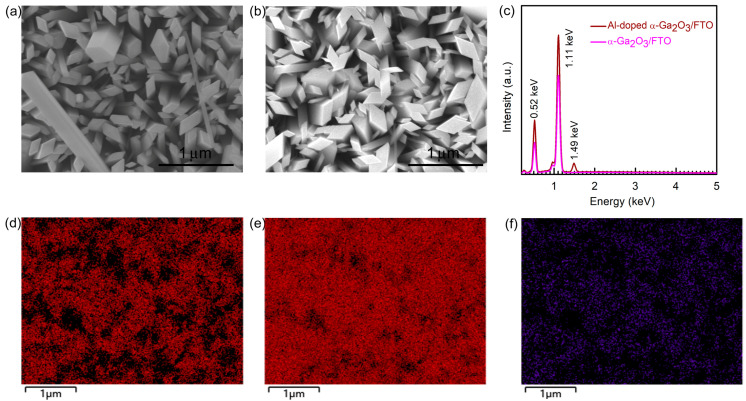
Surface morphology images of an undoped α-Ga_2_O_3_ nanorod array/FTO (**a**) and Al doped α-Ga_2_O_3_ nanorod array/FTO (**b**). (**c**) The EDS spectra of the undoped α-Ga_2_O_3_ nanorod array/FTO and Al-doped α-Ga_2_O_3_ nanorod array/FTO. EDS mapping images of O (**d**), Ga (**e**) and Al (**f**) in the Al-doped α-Ga_2_O_3_ nanorod array/FTO. The scale bars are 1 μm in (**a**–**f**).

Figure 1a,b, respectively, shows a surface morphology picture of an undoped α-Ga_2_O_3_ nanorod array/FTO and Al-doped α-Ga_2_O_3_ nanorod array/FTO structure by SEM. From the surface morphology images, the Al-doped α-Ga_2_O_3_ nanorod in Figure 1b also exhibits a diamond-like shape, which is similar to the α-Ga_2_O_3_ nanorod array without doping, as shown in Figure 1a. Therefore, Al doping did not obviously change the surface morphology of the α-Ga_2_O_3_ nanorod. As seen in Figure 1c, the EDS measurement obviously indicates the two dominated peaks at 0.52 keV and 1.11 keV for the undoped α-Ga_2_O_3_ nanorod array which, respectively, stand for the O and Ga elements. For the Al-doped α-Ga_2_O_3_ nanorod array, another EDS peak at 1.49 keV was also observed, corresponding to Al element. The EDS results also indicate an atom ratio of 5.57:1 for Ga: Al in the Al-doped α-Ga_2_O_3_ nanorod array. To further evaluate the element distributions in the Al-doped α-Ga_2_O_3_ nanorod array, Figure 1d–f are, respectively, the EDS mapping pictures of O, Ga, and Al element distributions in the Al-doped α-Ga_2_O_3_ nanorod array, in detail. From Figure 1d–f, O, Ga, and Al elements are uniformly distributed in the Al-doped α-Ga_2_O_3_ nanorod array. As a result, Al was successfully and uniformly doped into α-Ga_2_O_3_ nanorods.

**Figure 2:**
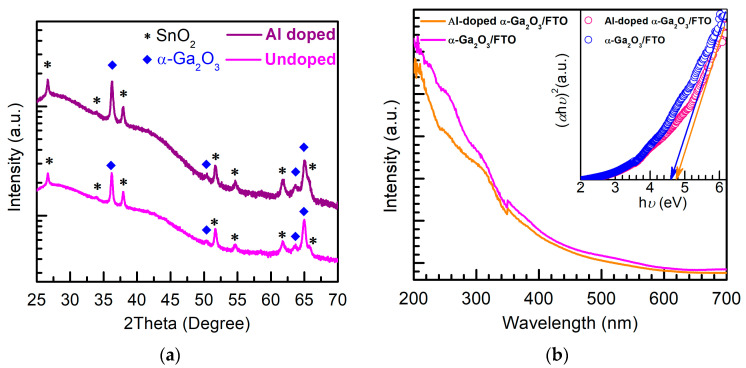
(**a**) XRD of an undoped α-Ga_2_O_3_ nanorod array/FTO (Undoped) and Al-doped α-Ga_2_O_3_ nanorod array/FTO (Al doped). (**b**) UV-vis diffuse reflectance absorption spectra of the undoped α-Ga_2_O_3_ nanorod array/FTO and Al-doped α-Ga_2_O_3_ nanorod array/FTO. The inset is the plots of (*αhν*)^2^ vs. *hν*.

The XRD measurements of an undoped α-Ga_2_O_3_ nanorod array/FTO and Al-doped α-Ga_2_O_3_ nanorod array/FTO were investigated to reveal the crystal structure, as shown in Figure 2a. The FTO substrate shows the diffraction peaks at ~26.6°, ~34°, ~37.9°, ~51.7°, ~54.7°, ~61.8° and ~65.9°, which can be attributed to be the SnO_2_ diffraction of (110), (101), (200), (211), (220), (310) and (301) faces, respectively. For the undoped α-Ga_2_O_3_ nanorod array/FTO, ~36.21°, ~50.35, ~63.69° and ~64.99° correspond to the (110), (024), (214) and (300) face diffractions of α-Ga_2_O_3_, respectively. Moreover, Al-doped α-Ga_2_O_3_ diffraction peaks are located at ~36.25°, ~50.45°, ~63.71°and ~65.07°, which also correspond to the (110), (024), (214), and (300) face diffractions of α-Ga_2_O_3_ [18,19], respectively. Owing to the smaller ionic radius of Al^3+^ (0.0535 nm) than that of Ga^3+^ (0.062 nm), the corresponding diffraction peaks of the Al-doped α-Ga_2_O_3_ nanorod array slightly shift to a larger angle in comparison with the undoped α-Ga_2_O_3_ nanorod array [18,19,24]. Figure 2b is the UV-vis diffuse reflectance absorption spectra of the undoped α-Ga_2_O_3_ nanorod array/FTO and Al-doped α-Ga_2_O_3_ nanorod array/FTO. After Al doping, a slightly blue-shift property of the Al-doped α-Ga_2_O_3_ nanorod array in the solar-blind UV region has been observed in Figure 2b. Based on the absorption properties, optical bandgaps can be estimated by extrapolating the straight-line portion to the *hν* axis near absorption edge, as shown in the inset of Figure 2b. The optical bandgap of the undoped α-Ga_2_O_3_ nanorod array is around ~4.6 eV, while the optical bandgap of the Al-doped α-Ga_2_O_3_ nanorod array is around ~4.8 eV. Due to the large bandgap of Al_2_O_3_ than that of Ga_2_O_3_, the slightly enlarged optical bandgap may originate from the incorporation of Al into α-Ga_2_O_3_.

**Figure 3:**
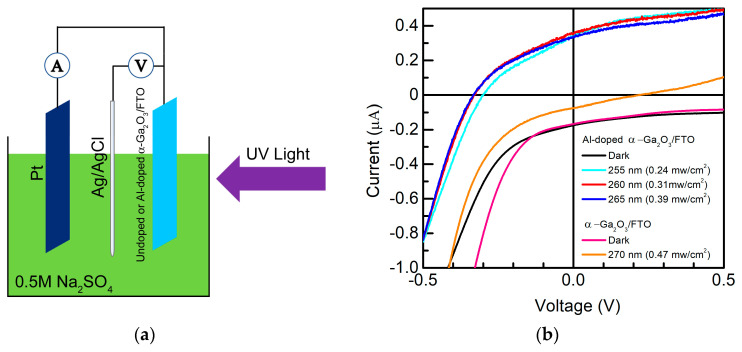

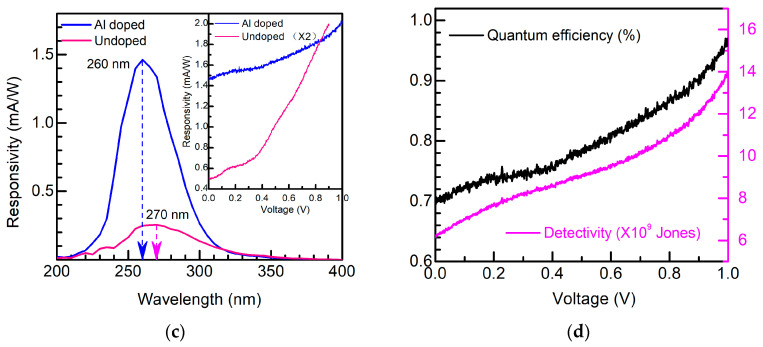
(**a**) Schematic depiction of the measurement setup in a PEC cell, where the undoped or Al-doped α-Ga_2_O_3_ nanorod array/FTO is selected as a photoanode. (**b**) Dark current and light current of the undoped α-Ga_2_O_3_ nanorod array/FTO device under 270 nm (0.47 mW/cm^2^) UV light illumination. Dark and light currents under 255 nm (0.24 mW/cm^2^), 260 nm (0.31 mW/cm^2^), and 265 nm (0.39 mW/cm^2^) UV light illumination of the Al-doped α-Ga_2_O_3_ nanorod array/FTO device. (**c**) Responsivity of the α-Ga_2_O_3_ nanorod array/FTO device (Undoped) and Al-doped α-Ga_2_O_3_ nanorod array/FTO device (Al doped) at 0 V (vs. Ag/AgCl). The inset shows the peak responsivity of the undoped and Al-doped devices varying with applied voltages (vs. Ag/AgCl). (**d**) Quantum efficiency and detectivity of the Al-doped α-Ga_2_O_3_ nanorod array/FTO device. ×2 represents the peak responsivity of the undoped α-Ga_2_O_3_ nanorod array/FTO device was multiplied by 2.

Figure 3a schematically illustrates the measurement setup during UV photodetection, where the undoped α-Ga_2_O_3_ nanorod array/FTO or Al-doped α-Ga_2_O_3_ nanorod array/FTO were selected as a photoanode in the shown PEC cell. Based on an electrochemical workstation, the three-electrode system additionally consists of Pt (counter electrode) ang Ag/AgCl (reference electrode). A total of 0.5 M Na_2_SO_4_ aqueous solution was used as the electrolyte. UV light was illuminated on the surface of the undoped α-Ga_2_O_3_ nanorod array/FTO or Al-doped α-Ga_2_O_3_ nanorod array/FTO, to evaluate the photoresponse properties. When the spectral responsivity was collected, the undoped α-Ga_2_O_3_ nanorod array/FTO or Al-doped α-Ga_2_O_3_ nanorod array/FTO, and Pt electrodes were connected with a lock-in amplifier.

Figure 3b shows the dark current and light current of an undoped α-Ga_2_O_3_ nanorod array/FTO device under 270 nm (0.47 mW/cm^2^) UV light illumination, and the dark and light currents under 255 nm (0.24 mW/cm^2^), 260 nm (0.31 mW/cm^2^), and 265 nm (0.39 mW/cm^2^) UV light illumination of an Al-doped α-Ga_2_O_3_ nanorod array/FTO device. The light current increases from −0.17 mA to −0.08 mA at 0 V (vs. Ag/AgCl) under 270 nm illumination for the undoped α-Ga_2_O_3_ nanorod array/FTO device. Obviously, the light current of the Al-doped α-Ga_2_O_3_ nanorod array/FTO device increases from −0.18 mA to 0.34 mA at 0 V (vs. Ag/AgCl) under 255 nm illumination, from −0.18 mA to 0.35 mA at 0 V (vs. Ag/AgCl) under 260 nm illumination, and from −0.18 mA to 0.33 mA at 0 V (vs. Ag/AgCl) under 265 nm illumination. And larger net photocurrent was realized after Al was doped into the α-Ga_2_O_3_ nanorod array. The net short-circuit photocurrent produced at 0 V (vs. Ag/AgCl) indicates that our devices can realize the self-powered photodetection for both the undoped α-Ga_2_O_3_ nanorod array/FTO device and the Al-doped α-Ga_2_O_3_ nanorod array/FTO device. Additionally, the open-circuit voltage is estimated to be ~−0.3 V under 255 nm illumination, ~−0.33 V under 260 nm illumination, and ~−0.33 V under 265 nm illumination for the Al-doped α-Ga_2_O_3_ nanorod array/FTO device. Considering the undoped α-Ga_2_O_3_ nanorod array/FTO and Al-doped α-Ga_2_O_3_ nanorod array/FTO structure as photoanodes, the light current shifting towards the positive direction compared with the negative dark current indicates that positive photocurrent has been generated. Thus, the photogenerated electrons flows from undoped α-Ga_2_O_3_ nanorod array or Al-doped α-Ga_2_O_3_ nanorod array to FTO in the undoped α-Ga_2_O_3_ nanorod array/FTO or Al-doped α-Ga_2_O_3_ nanorod array/FTO device at 0V (vs. Ag/AgCl). The detailed mechanism will be explained in the working mechanism part. Figure 3c shows solar-blind UV photoresponse spectra at 0 V (vs. Ag/AgCl). The peak wavelength is ~270 nm, with a peak responsivity of ~0.25 mA/W for the undoped α-Ga_2_O_3_ nanorod array/FTO device, while the peak wavelength is 260 nm with a peak responsivity of ~1.46 mA/W for the Al-doped α-Ga_2_O_3_ nanorod array/FTO device. Because of the larger optical bandgap of the Al-doped α-Ga_2_O_3_ nanorod array, the peak photoresponse shifts to the shorter wavelength, which is consistent with the slight blue-shift property of the Al-doped α-Ga_2_O_3_ nanorod array in the solar-blind UV region in Figure 2b. And the enhanced responsivity is also related to Al doping. According to the literature, the photoresponse of Ga_2_O_3_ can be improved after Al doping, although further investigations should be conducted to reveal the reason [12,13,14,15,16]. Herein, the Al-doped α-Ga_2_O_3_ nanorod array is also ~5.84 times larger in peak responsivity than the undoped α-Ga_2_O_3_ nanorod array at 0 V (vs. Ag/AgCl). The inset of Figure 3c shows the peak responsivity of the undoped α-Ga_2_O_3_ nanorod array/FTO device and Al-doped α-Ga_2_O_3_ nanorod array/FTO device varying with the applied voltages (vs. Ag/AgCl). The peak responsivity increases from ~0.25 mA/W at 0 V to 1 mA/W at 0.9 V for the undoped α-Ga_2_O_3_ nanorod array/FTO device. Obviously, the peak responsivity increases from ~1.46 mA/W at 0 V to 2.02 mA/W at 1 V for the Al-doped α-Ga_2_O_3_ nanorod array/FTO device, which is much larger than that of the undoped device. Based on the responsivity and dark current, the quantum efficiency and detectivity of the Al-doped α-Ga_2_O_3_ nanorod array/FTO device can be roughly evaluated [5]. Figure 3d shows the voltage dependences of the calculated quantum efficiency and detectivity in details. The quantum efficiency varies from ~0.7% at 0 V (vs. Ag/AgCl) to ~0.96% at 1 V (vs. Ag/AgCl), while the detectivity increases from ~6 × 10^9^ Jones at 0 V (vs. Ag/AgCl) to ~1 × 10^10^ Jones at 1 V (vs. Ag/AgCl). The enhanced responsivity, quantum efficiency, and detectivity are ascribed from the wider depletion region when positive voltages are added to the FTO electrode.

**Figure 4:**
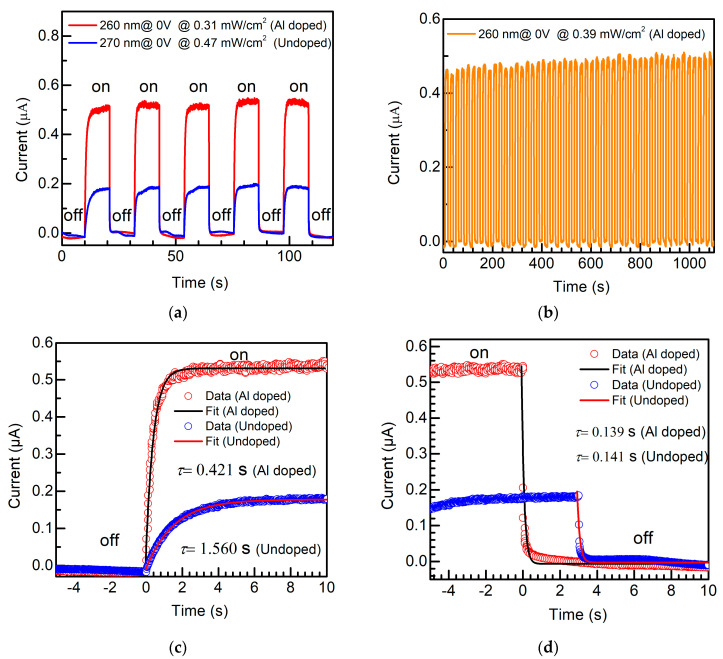
(**a**) Time-dependent current of an undoped α-Ga_2_O_3_ nanorod array/FTO device (Undoped) under 270 nm light illumination (0.47 mW/cm^2^), and Al-doped α-Ga_2_O_3_ nanorod array/FTO device (Al doped) under 260 nm light illumination (0.31 mW/cm^2^), in 5 cycles, with 10 s on and 10 s off. (**b**) Time-dependent current of the Al-doped α-Ga_2_O_3_ nanorod array/FTO device in 50 cycles, with 10 s on and 10 s off, under 260 nm light illumination (0.39 mW/cm^2^). (**c**) Transient photoresponse curves of the α-Ga_2_O_3_ nanorod array/FTO device (Undoped), and Al-doped α-Ga_2_O_3_ nanorod array/FTO device (Al doped), during rise processes in (**a**). (**d**) Transient photoresponse curves of the α-Ga_2_O_3_ nanorod array/FTO device (Undoped), and Al-doped α-Ga_2_O_3_ nanorod array/FTO device (Al doped), during decay processes in (**a**). The α-Ga_2_O_3_ nanorod array/FTO device and Al-doped α-Ga_2_O_3_ nanorod array/FTO device were performed as photoanodes in a PEC cell at 0 V (vs. Ag/AgCl).

As shown in Figure 4a, when the photoanode in a PEC cell was 0 V (vs. Ag/AgCl), we, respectively, measured the time-dependent current curves of an undoped α-Ga_2_O_3_ nanorod array/FTO under 270 nm light illumination (0.47 mW/cm^2^) and Al-doped α-Ga_2_O_3_ nanorod array/FTO under 260 nm light illumination (0.31 mW/cm^2^), with 10 s on and 10 s off. Thus, our device shows good stability and repetition at the self-powered photodetection mode in 5 cycles. And the steady light current of the Al-doped α-Ga_2_O_3_ nanorod array/FTO is also much larger than that of the undoped α-Ga_2_O_3_ nanorod array/FTO. The rise processes of the undoped α-Ga_2_O_3_ nanorod array/FTO are clearly longer than those of the Al-doped α-Ga_2_O_3_ nanorod array/FTO. When the shutter is off, the light current immediately drops to the initial level for both of the devices. To further evaluate long-period stability and repetition, we also measured a time-dependent current curve of the Al-doped α-Ga_2_O_3_ nanorod array/FTO under 260 nm light illumination (0.39 mW/cm^2^), with 10 s on and 10 s off, at the self-powered photodetection mode in 50 cycles. As shown in Figure 4b, the steady light current of the Al-doped α-Ga_2_O_3_ nanorod array/FTO nearly remains the same value at 0 V (vs. Ag/AgCl) under 260 nm illumination (0.39 mW/cm^2^). By the following equation: *I*(*t*) = *I*_s_ + *Ie*^−*t*/*τ*^ (where *I*(*t*) is the light current decay, *I*_s_ is the steady current, *I* is the photocurrent, and *τ* is the rise time or decay time), the rise time and decay time can be fitted during a rise process and a decay process, respectively, in Figure 4a. Figure 4c is the transient photoresponse of the undoped α-Ga_2_O_3_ nanorod array/FTO under 270 nm light illumination (0.47 mW/cm^2^) and Al-doped α-Ga_2_O_3_ nanorod array/FTO under 260 nm light illumination (0.31 mW/cm^2^) at 0 V (vs. Ag/AgCl) during rise processes. From Figure 4c, the rise time of the undoped α-Ga_2_O_3_ nanorod array/FTO device and Al-doped α-Ga_2_O_3_ nanorod array/FTO device is, respectively, 1.560 s and 0.421 s. The longer rise time is consistent with the slower rise process in Figure 4a. Figure 4d is the corresponding transient photoresponse curves during decay processes in Figure 4a. The decay time of the undoped α-Ga_2_O_3_ nanorod array/FTO device and Al-doped α-Ga_2_O_3_ nanorod array/FTO device is fitted to be 0.141 s and 0.139 s, respectively. Unlike the much faster rise time of the Al-doped α-Ga_2_O_3_ nanorod array/FTO device (Figure 4c), the decay time is nearly the same for both of the devices.

**Figure 5:**
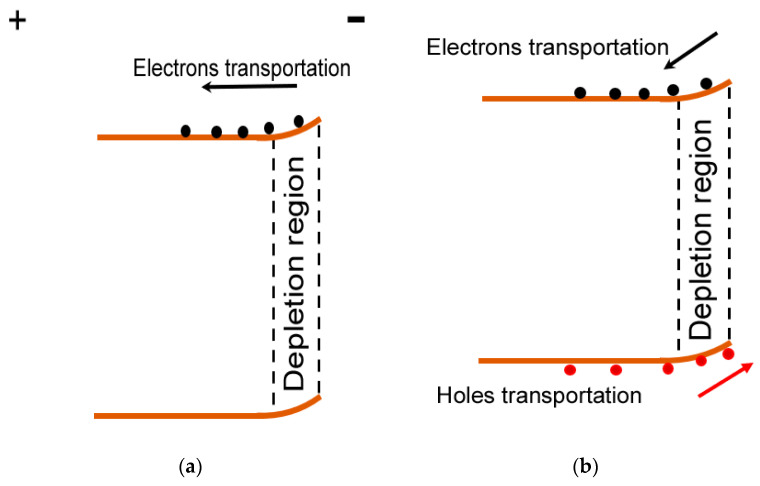

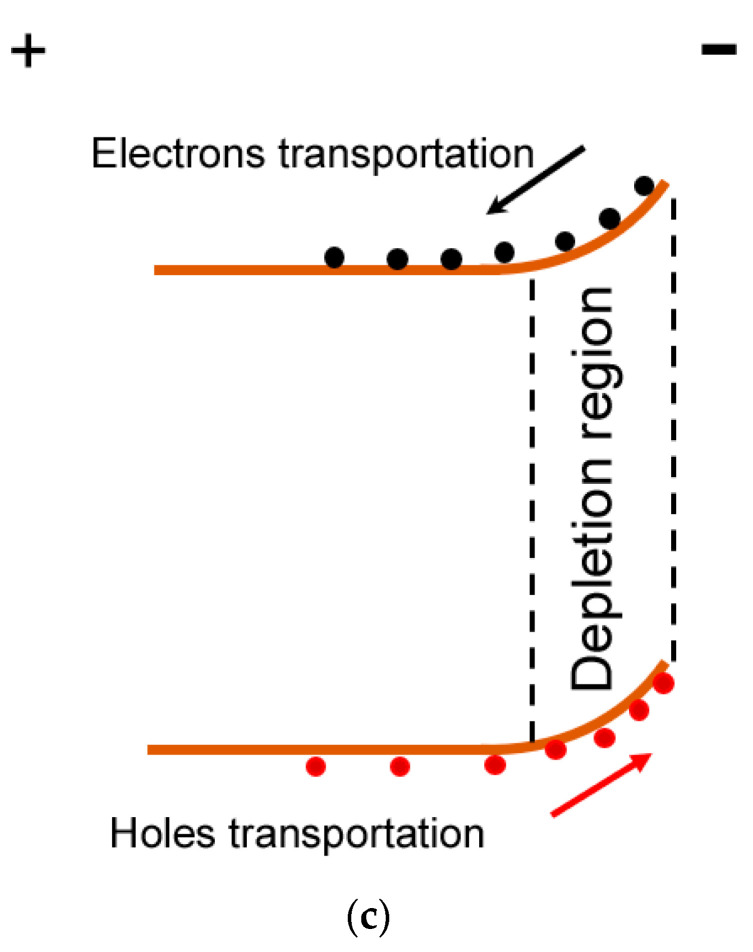
(**a**) Energy band diagram of an undoped α-Ga_2_O_3_ nanorod array/FTO or Al-doped α-Ga_2_O_3_ nanorod array/FTO device as photoanodes in a PEC cell. (**b**) Energy band diagram of the undoped α-Ga_2_O_3_ nanorod array/FTO or Al-doped α-Ga_2_O_3_ nanorod array/FTO device at 0 V (vs. Ag/AgCl) under solar-blind UV illumination. (**c**) Energy band diagram of the undoped α-Ga_2_O_3_ nanorod array/FTO or Al-doped α-Ga_2_O_3_ nanorod array/FTO device at positive biases (vs. Ag/AgCl) under solar-blind UV illumination.

To further explain the working mechanism, Figure 5 schematically depicts energy band diagrams of the undoped α-Ga_2_O_3_ nanorod array/FTO device or Al-doped α-Ga_2_O_3_ nanorod array/FTO device as a photoanode in the PEC cell. In the dark, a Schottky junction is produced between the nanorod array and Na_2_SO_4_ electrolyte when a nanorod array on FTO is immersed into a Na_2_SO_4_ aqueous solution in Figure 5a [20]. Thus, a depletion region is generated at the surface of the nanorod array. Owing to the nanorod array/FTO structure selected as the photoanode; positive biases are added to FTO when the PEC cell works as shown in Figure 5a. Therefore, the forward current in our device is just the reverse current as in the traditional Schottky junction. However, photogenerated electron-hole pairs are produced in the depletion region of the undoped α-Ga_2_O_3_ nanorod array or Al-doped α-Ga_2_O_3_ nanorod array under solar-blind UV light illumination in Figure 5b,c. Under the drift of the built-in electric field, photogenerated electrons move from the undoped α-Ga_2_O_3_ nanorod array or Al-doped α-Ga_2_O_3_ nanorod array to FTO, while photogenerated holes moves from the undoped α-Ga_2_O_3_ nanorod array or Al-doped α-Ga_2_O_3_ nanorod array to the electrolyte. Thus, a large positive photocurrent is generated under solar-blind UV light illumination, the direction of which is the same transportation direction as that of the forward dark current. Thus, the light current shifting towards a positive direction has been observed for both the undoped α-Ga_2_O_3_ nanorod array/FTO device and Al-doped α-Ga_2_O_3_ nanorod array/FTO devices in Figure 3 and Figure 4. It is noteworthy that the larger bandgap of the Al-doped α-Ga_2_O_3_ may lift its conduction edge as the incorporation of MgO into ZnO [25]. Therefore, a larger Schottky barrier may exist in the Al-doped α-Ga_2_O_3_ nanorod array/FTO PEC cell than that in the undoped α-Ga_2_O_3_ nanorod array/FTO PEC cell. As a result, a wider depletion region in the Al-doped α-Ga_2_O_3_ nanorod array/FTO PEC cell can improve the separation of more photogenerated electron-hole pairs, and contributes to a larger photocurrent and responsivity. Therefore, the larger photocurrent and responsivity have been demonstrated through doping Al into the α-Ga_2_O_3_ nanorod array. Generally, the depletion region of a Schottky junction can be enhanced under reverse biases. Considering the undoped α-Ga_2_O_3_ nanorod array/FTO or Al-doped α-Ga_2_O_3_ nanorod array/FTO as the photoanode in the PEC cell, such biases are the forward biases as indicated in Figure 5a. As a result, the depletion region becomes wider in Figure 5c under positive biases than that at 0 V in Figure 5b. Therefore, larger photocurrent is formed under positive biases, which is also the reason of the improved responsivity, quantum efficiency, and detectivity when positive voltages are added to the FTO electrode, as shown in Figure 3c,d.

## 4. Conclusions

In this work, using a simple and cheap processes, including hydrothermal and post-annealing, we realized an Al-doped α-Ga_2_O_3_ nanorod array on FTO. To the best of our knowledge, it is the first time that Al-doped α-Ga_2_O_3_ nanorod array has been fabricated on FTO via a much simpler and cheaper way than that based on MOCVD, magnetron sputtering, MBE and PLD. When the Al-doped α-Ga_2_O_3_ nanorod array/FTO structure is served as a photoanode in a PEC cell, the Al-doped α-Ga_2_O_3_ nanorod array/FTO photodetector had the self-powered photodetection properties at 0 V (vs. Ag/AgCl) with a peak responsivity of ~1.46 mA/W at 260 nm. The rise time was 0.421 s, and the decay time was 0.139 s, under solar-blind UV (260 nm) illumination. Compared with the undoped α-Ga_2_O_3_ nanorod array/FTO device, the peak responsivity of the Al-doped device was ~5.84 times larger, and the response speed was relatively faster. The reason is that a larger Schottky barrier and wider depletion region may exist in the Al-doped α-Ga_2_O_3_ nanorod array/FTO PEC cell than that in the undoped α-Ga_2_O_3_ nanorod array/FTO PEC cell. As a result, the wider depletion region in the Al-doped α-Ga_2_O_3_ nanorod array/FTO PEC cell can improve the separation of more photogenerated electron-hole pairs, contributing to a larger photocurrent and responsivity. The peak responsivity of the Al-doped α-Ga_2_O_3_ nanorod array/FTO device increased to 2.02 mA/W at 1 V (vs. Ag/AgCl). Additionally, with the increase of the positive biases, the enlarged depletion region contributes to the enhanced responsivity, quantum efficiency (from ~0.7% at 0 V (vs. Ag/AgCl) to ~0.96% at 1 V (vs. Ag/AgCl)), and detectivity (from ~6 × 10^9^ Jones at 0 V (vs. Ag/AgCl) to ~1 × 10^10^ Jones at 1 V (vs. Ag/AgCl)). Therefore, doping Al into α-Ga_2_O_3_ may provide a route to enhance the self-powered photodetection performances of α-Ga_2_O_3_ nanorod arrays.

## Data Availability

The data are available upon reasonable request from the authors.
